# Sex-independent neuroprotection with minocycline after experimental thromboembolic stroke

**DOI:** 10.1186/2040-7378-3-16

**Published:** 2011-12-16

**Authors:** Md Nasrul Hoda, Weiguo Li, Ajmal Ahmad, Safia Ogbi, Marina A Zemskova, Maribeth H Johnson, Adviye Ergul, William D Hill, David C Hess, Irina Y Sazonova

**Affiliations:** 1 Department of Neurology, Georgia Health Sciences University, Augusta, GA, USA; 2 Department of Physiology, Georgia Health Sciences University, Augusta, GA, USA; 3 Department of Medicine, Georgia Health Sciences University, Augusta, GA, USA; 4 Department of Biostatistics, Georgia Health Sciences University, Augusta, GA, USA; 5 Department of Cell Biology and Anatomy, Georgia Health Sciences University, Augusta, GA, USA; 6 Charlie Norwood VA Medical Center, Augusta, GA, USA

**Keywords:** thromboembolic stroke, animal model, neuroprotection, minocycline, sex, MMP-9

## Abstract

**Background:**

Minocycline provides neurovascular protection reducing acute cerebral injury. However, it is unclear whether minocycline is effective in females. We tested minocycline in both sexes and aged animals using a novel embolic stroke model in mice that closely mimics acute thromboembolic stroke in humans.

**Methods:**

Five groups of mice were subjected to thromboembolic stroke: adult males, aged males, adult females, aged females, and adult ovariectomized females. They were treated with phosphate saline (vehicle) or minocycline (6 mg/kg) immediately after stroke onset. Behavioral outcomes, infarct volumes and cerebral blood flow were assessed. The effect of minocycline on expression and activity of MMP-9 was analyzed.

**Results:**

The model resulted in reproducible infarct in the experimental groups. As expected, adult females were significantly more resistant to cerebral ischemic injury than males. This advantage was abolished by aging and ovariectomy. Minocycline significantly reduced the infarct volume (P < 0.0001) and also improved neurologic score (P < 0.0001) in all groups. Moreover, minocycline treatment significantly reduced mortality at 24 hours post stroke (P = 0.037) for aged mice (25% versus 54%). Stroke up-regulated MMP-9 level in the brain, and acute minocycline treatment reduced its expression in both genders (P < 0.0001).

**Conclusion:**

In a thromboembolic stroke model minocycline is neuroprotective irrespective of mouse sex and age.

## Introduction

Interest in sex differences during acute stroke is an area of growing interest. A consistent finding in rodent models of cerebral ischemia is that young females have smaller infarct sizes and better outcomes than young male rodents [1]. This female protection is lost after ovariectomy. However, the sex difference in stroke is only present when the brain is reperfused; in permanent occlusion the sex difference vanishes [2]. Moreover, in older rodents, the sex difference seen in younger animals is lost [3]. Reproductively senescent older female and male mice have similar infarct sizes after 2 hours of ischemia and 22 hours of reperfusion [4].

The effect of sex on stroke outcome may also be hormone independent [3]. Recent studies suggest the existence of sex-divergent cell death pathways operating during cerebral ischemia [5]. The neuronal nitric oxide (NO)/Poly ADP ribose (PARP) pathways appear to only mediate cell death during cerebral ischemia in male rodents [5]. These sexually divergent pathways may influence how females and males respond to acute stroke treatments. For example, PARP inhibitors, and inhibitors of neuronal NOS are reported to be only neuroprotective in male mice [5,6]. This concern over sex-related effects has resulted in recommendations from the Stroke Academic Industry Roundtable to include female animals and older animals in pre-clinical testing [7].

The choice of experimental stroke model is also important. While the suture occlusion model is often used for both reperfusion and permanent ischemic models, a suture is an unnatural occlusion mechanism and reperfusion in human stroke is seldom achieved as abruptly as removal of the suture in an animal. This abrupt reperfusion may modify the cellular consequences of the ischemic process [8,9]. An embolic clot model better models the human clinical condition [10,11]. Moreover, mechanical and thrombolytic reperfusions have different profiles and time courses of cerebral blood flow (CBF) and barrier damage [12]. It is also important to test adult and older rodents of both sexes in embolic clot occlusion models where mostly young male rodents have been used to date.

Minocycline, a tetracycline derivative, is a promising neuroprotective drug that has reduced infarct size and improved functional outcomes in multiple experimental models [13-19]. Recent evidence suggests that it is also promising in clinical trials [20,21]. Minocycline inhibits PARP-1 at nanomolar concentrations, but has multiple mechanisms of action including inhibition of MMP-9 [14,15,22,23]. However, in a suture occlusion model, minocycline reduced infarct size in male mice, but not in recently ovariectomized (OVX) female mice [5,24,25]. In this study we used a new thromboembolic model with spontaneous reperfusion with a humanized clot. Our aims were to determine if minocycline was neuroprotective in an embolic clot model in mice of both sexes and in aged mice. We also utilized more than 12 weeks interval between ovariectomy in adult females and embolic stroke to mimic estrogen homeostasis post-menopause in humans. A secondary aim was to determine if there was a sex-specific change in MMP-9 levels.

## Materials and methods

### Animals

All the experimental procedures have been approved by the Institutional Animal Care and Use Committee (IACUC) of Georgia Health Sciences University (GHSU) in accordance with NIH *Guide for the Care and Use of Laboratory Animals*. Wild type C57BL/6J male and female mice were purchased from the Jackson Laboratory (Bar Harbor, Maine) and housed in the GHSU Animal Facility approved by the American Association for Accreditation of Laboratory Animal Care. Female mice were ovariectomized at 11-12 weeks of age in the Jackson Laboratory and were about 12 weeks post ovariectomy prior to stroke. The mean ages of stroked adult animals were: 24.0 ± 4.6 weeks for adult males, 22.9 ± 3.3 weeks for adult females, and 23.9 ± 2.7 weeks for OVX females. The aged C57BL/6 animals (18.1 ± 0.8 month males and 16.0 ± 1.1 month females) were from GHSU in-house breeding.

### Estrus cycle analysis in mice

Five weeks after ovariectomy female mice (n = 7) were tested for estrous cyclicity to confirm loss of estrogenic effect and simulation of postmenopausal stage. The vaginal smears were compared to cycling adult females (n = 7) and aged females (n = 5). All vaginal smears were obtained daily between 9:00 am and 1:00 pm for 8 weeks and stained with 2% Giemsa solution (Sigma) as described [26].

### Estrogen levels

Plasma samples (50 μl) from all post-stroke survived females and eight randomly selected adult male mice were used to measure estrogen by enzyme immunoassay (Cayman Chemical, Ann Arbor, MI).

### Preparation of emboli

The method of clot preparation was adapted from earlier reports [10,27] and modified to increase the strength of uniformity of the fibrin rich core and stability of occlusion. Briefly, mouse arterial blood was supplemented with human fibrinogen (2 mg/mL), and immediately clotted in PE-50 tubing for 6 hours at room temperature followed by storage at 4°C. Before use, the clot (~5 cm) was transferred into a modified PE-10 tube filled with sterile saline and retracted. The clot was then transferred to a Petri dish containing phosphate-buffered saline and left for further retraction at room temperature for 4 hours. A single 9.0 ± 0.5 mm long clot was transferred to a modified PE-10 catheter for embolization.

### Thromboembolic stroke model

Mice were anesthetized with 3.5% isofluorane and maintained with 2.0% during the surgery. Body temperature was maintained at 37°C by a thermo-regulated surgery pad. By a midline incision on the ventral side of the neck, the right common carotid artery, the right external carotid artery (ECA), and the right internal carotid artery (ICA) were assessed [10]. A modified PE-10 catheter containing a clot was introduced into the ECA lumen through a small hole, advanced into the ICA, and the clot was gently injected with 100 μL of the sterile phosphate-saline buffer (PBS). After thromboembolization the catheter was removed immediately. To identify the location of an embolus after injection, the fibrin-rich clot was labeled by Evans blue before injection [10]. In the sham group, an equal volume of PBS without clot was delivered. Occlusion was confirmed by ≥70% drop in cerebral blood flow (CBF) compared to the pre-ischemic value. Animals that showed sustained occlusion were included. The success rate of thromboembolic MCA occlusion was 95% (208 successes from 219 total stroked animals) based on changes in CBF. Animals were randomized immediately after clot injection and treated with either phosphate saline (PBS, vehicle) or minocycline (Sigma; 6 mg/kg) via bolus IV injection to tail vein (0.1 mL/10 g body weight). Sham-operated mice served as controls.

### Regional cortical laser-doppler flowmetry

Cortical laser-Doppler flowmetry ([LDF], Perimed. Inc.) was performed 30 min and 3 min before occlusion to record a consistent basal level of peripheral blood flow in the middle cerebral artery region, and also recorded during occlusion [28]. For this purpose, shallow indentation was made in the parietal skull (A - P 2 mm, and lateral 3 mm with respect to bregma) with a low-speed drill for placement of the LDF probe holder (PH07-6, Perimed. Inc.). The LDF signal was recorded semi-continuously and averaged over 10-minute intervals for each time point.

### Neurological assessment

Neurologic deficits in the animals were assessed at 24 hr post stroke by a 5-point scale scoring: 0, no deficit; 1, forelimb flexion deficit on contralateral side; 2, decreased resistance to lateral push and torso turning to the ipsilateral side when held by tail; 3, very significant circling to affected side and reduced capability to bear weight on the affected side; 4, rarely moves spontaneously and prefer to stay in rest.

### Infarct analysis

Mice were euthanized at 24 hours after stroke for injury assessment. Brains were perfused with ice cold 0.01 M phosphate-buffered saline (PBS), cut into 1-mm coronal slices. Every other slice was stained with 2% 2,3,5-triphenyltetrazolium chloride (TTC, Sigma) for 30 minutes at 37°C, then fixed with 10% formalin in PBS. The images were digitalized and the infarct volume was analyzed by software SPOT Advanced (Sterling Heights, MI) as previously described [14,28,29]. The infarct volumes were quantified as both direct volume (in mm^3^) and indirect volume (percent volume of the total ischemic hemisphere). The measurement of infarct size was made by an investigator blinded to treatment group.

### Immunoblotting

Six hours after the stroke onset, mice were euthanized, and brains were perfused with cold phosphate-buffered saline and extracted. Ischemic hemisphere tissue was homogenized and lysed in complete Lysis-M EDTA-free buffer (Roche Diagnostics, Indianapolis, IN). The amount of total protein was quantified using the EZQ^® ^Protein Quantitation Kit (Invitrogen). Samples (30 micrograms of total protein) were subjected to SDS-PAGE using 10% NuPAGE^® ^Novex^® ^Bis-Tris gels (Invitrogen) and transferred to 0.2 μm PVDF membranes (Millipore, Billerica, MA). The membrane was blocked by for non-specific binding (5% BSA solution), and incubated with polyclonal anti-MMP-9 antibody (G6571, Cell Signaling Technology, Danvers, MA) at 4°C overnight, followed by HRP-conjugated donkey anti-rabbit IgG antibody (Jackson ImmunoResearch, West Grove, PA). Membranes were re-probed with mouse monoclonal anti-β-actin antibody (Sigma-Aldrich Co.) as a loading control. Proteins were visualized with the ECL detection system (Pierce, Thermo Fisher Scientific) on autoradiography film (Denville Scientific, Metuchen, NJ). The image was scanned and processed for densitometric measurement in Image-J software. The analysis of MMP-9 antigen was made by an investigator blinded to treatment group.

### Gelatin zymography

Basal plasma MMP activity, and at 6 hours after stroke, was detected using gelatin zymography [14,23,28,29] in duplicate for each sample. The ischemic hemisphere tissue was homogenized in 0.05 mol/L Tris buffer (pH 7.4) containing 0.1 mol/L NaCl and 0.17 ng/mL PMSF. Citrated blood samples were immediately centrifuged at 4000 rpm for 20 min at 4°C and total plasma protein was determined with the BCA assay (Pierce). The total lysed basal protein (100 micrograms) or plasma protein (30 micrograms) was loaded and separated by a 10% Tris-glycine gel with 0.1% porcine gelatin (Sigma). The gels were washed with renaturing buffer and incubated with developing buffer (BioRad Labs.) at 37°C for 20 hours. Finally, the gels were stained with Coomassie blue R-250 followed by appropriate destaining. The gelatinolytic activity of the samples was assessed by densitometric analysis (Gel-Pro v 3.1, Media Cybernetics, Carlsbad, CA) of the bands as a relative comparison to a standard band of recombinant enzyme. To minimize inter-gel variability, all gels had a control lane loaded with 0.5 ng recombinant enzyme, which was used as a standard optical density and enzyme amount (in ng). The density of the sample bands were expressed as maximal optical density relative to the standard band. The analysis of MMP-9 activity was made by an investigator blinded to treatment group.

Measurement of cerebral perfusion and MRI procedureare described in the Additional Methods.

### Statistical analysis

All data are expressed as mean ± SD. Statistical analyses were performed using SAS^® ^9.2 (SAS Institute, Inc., Cary, NC). Infarction and neurologic score were analyzed for males using a 2 Age (adult vs. aged) × 2 TRT (saline vs. minocycline) ANOVA and for females using a 3 Group (adult, aged, OVX) × 2 TRT (saline vs. minocycline) ANOVA. Adult males and females were compared using a 2 Sex (male vs. female) × 2 TRT (saline vs. minocycline) ANOVA. MMP-9 antigen and activity levels were analyzed using a 2 Sex (male vs. OVX) × 3 TRT (sham, stroke+vehicle, stroke+minocycline) ANOVA. In all analyses, interactions were tested for possible differential effects of minocycline treatment on age, sex or group. Tukey's multiple comparison tests were used to compare means for significant main effects. The effect of minocycline on mortality at 24 hours was analyzed in adult and aged mice using Fisher's Exact test. Group numbers are shown in parentheses. Statistical significance was determined at P < 0.05.

## Results

### Thromboembolic stroke model

**Figure 1:**
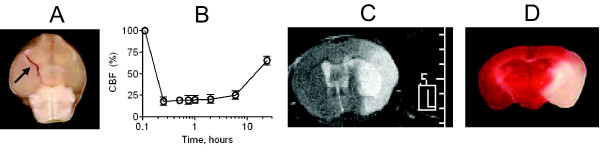
**Thromboembolic stroke model in C57BL/6 adult male mice**. A) The clot is inserted at the MCA origin. Clots were visualized with Evan's Blue dye (arrowhead). B) Regional cerebral blood flow (CBF) over time as measured with Laser Doppler flowmetry (means ± SD, n = 7). The CBF declines to 18 ± 4% of baseline following embolization, which was associated with a reproducible sized infarct of the MCA territory (115 ± 22 mm^3^) at 24 post stroke hours. C, D) Representative infarct area at 24 hours as determined by T2 diffusion-weighted MRI (C) and with 2,3,5-triphenyltetrazolium chloride staining of the coronal brain sections (D). Additional representative images of thromboembolic stroke model are shown in the Additional file 1 (Figure S1), Additional file 2 (Figure S2), Additional file 3 (Figure S3), Additional file 9 (Additional Figure Legends) and Additional file 10 (Additional Methods).

**Figure 2:**
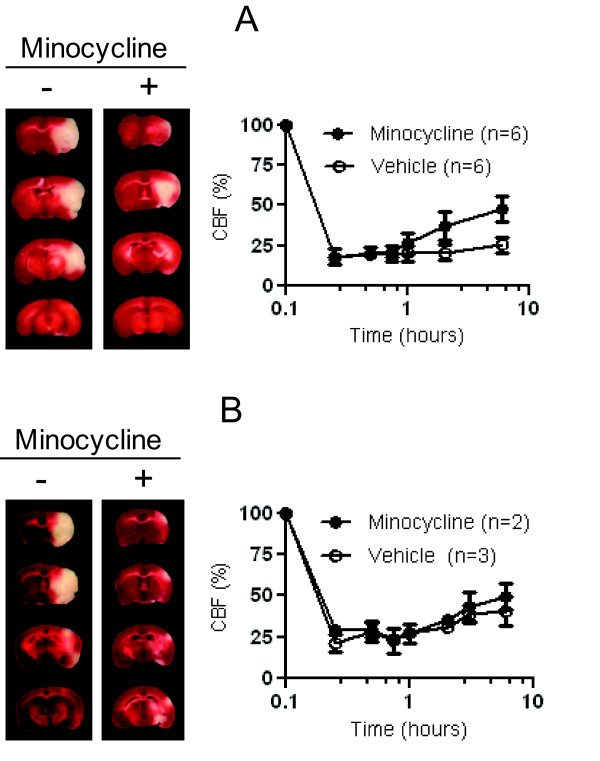
**Representative effect of minocycline in adult (A) and aged (B) males**. Coronal TTC sections, outlining the infarct area in representative subjects treated by the phosphate-saline buffer (vehicle) or minocycline at 24 hours, are showed in the left panels. The corresponding change of regional CBF during the first 6 hours after stroke is showed on the right panel. The value of CBF is presented as means ± SD.

**Figure 3:**
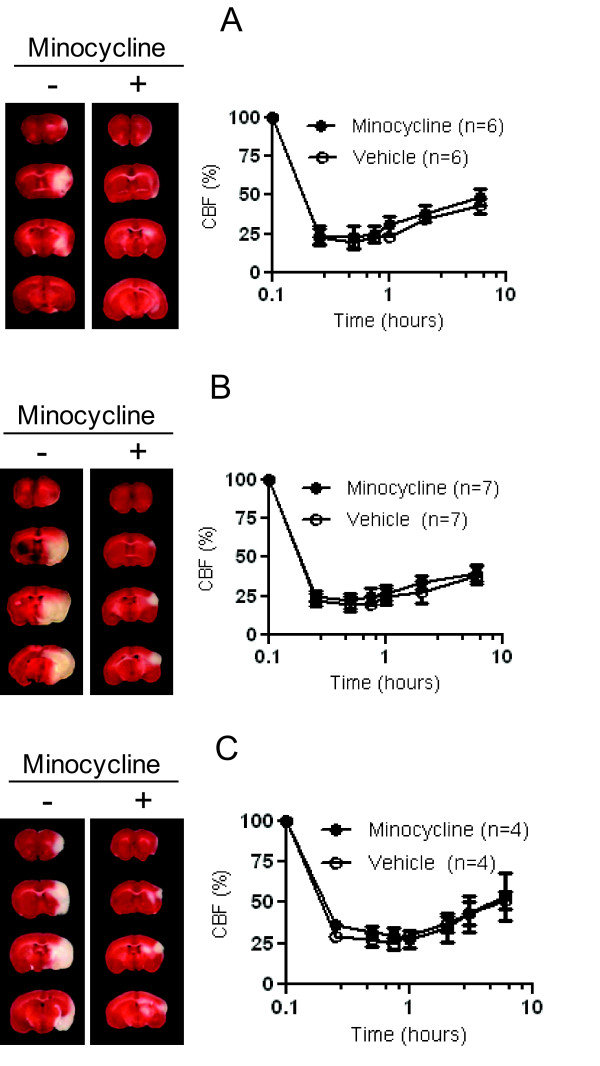
**Representative effect of minocycline in adult (A), retired (B), and OVX (C) females**. Coronal TTC section, outlining the infarct area in a representative subjects treated by vehicle or minocycline at 24 hours, are showed on the left panel. The corresponding change of regional CBF during the first 6 hours after stroke is showed on right panel. The value of CBF is presented as means ± SD.

The model was initially optimized using C57BL/6 wild type male mice (24 ± 4 weeks old, 25 - 30 g) by injecting a fibrin rich clot formed ex-vivo into the right MCA. Figure 1A represents the brain image with the clot occluding the ipsilateral MCA origin. The embolization led to consistent reduction of CBF to 18.1 ± 4.7% of baseline that persisted (25.4 ± 4.6%) for 6 hours (Figure 1B and Additional file 1, Figure S1). There was a slow spontaneous CBF restoration by 24 hours that reached 65 ± 5% from the baseline as determined by cortical laser-Doppler flowmetry (Figure 1B). Cerebral perfusion imaging with the PeriScan system yielded similar results (Additional file 2, Figure S2). Thromboembolic stroke resulted in reproducible infarct (115 ± 22 mm^3^, n = 14) which was confirmed by T2-weighted MRI (Figure 1C and Additional file 3, Figure S3) or coronal brain staining with 2,3,5-triphenyltetrazolium chloride (Figure 1D and Additional file 3, Figure S3). In this model adult males treated with saline had reliable injury and low mortality rate at 24 hours (3 out of 17 animals, Table 1). The model was applied to study the gender difference in neuroprotection with minocycline using various subgroups of male (Figure 2) and female (Figure 3) mice.

**Table 1:** Mortality rates at 24 hours after stroke

Group	Treatment	Total in studies, n	Dead, n (mortality rate)
Adult males	Vehicle	17	3 (17%)
	Minocycline	13	1 (8%)

Aged males	Vehicle	17	10 (59%)
	Minocycline	9	2 (22%)

Adult females	Vehicle	15	2 (13%)
	Minocycline	15	3 (20%)

Aged females	Vehicle	23	11 (48%)
	Minocycline	15	4 (27%)

OVX females	Vehicle	26	13 (50%)
	Minocycline	15	1 (7%)

### Acute minocycline treatment is neuroprotective for male mice

**Figure 4:**
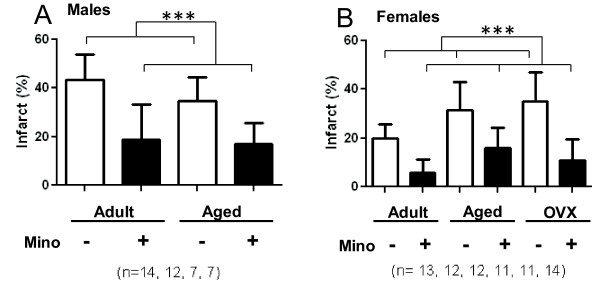
**Minocycline reduces brain tissue injury in males (A) and females (B)**. The infarct size of ischemic MCA territory was estimated as the percent volume of the total ischemic hemisphere. Regardless of treatment adult females had significantly smaller infarct volumes (both P < 0.0001, ***) than aged and OVX females who were not different than each other (P = 0.77). All data expressed as means ± SD.

**Figure 5:**
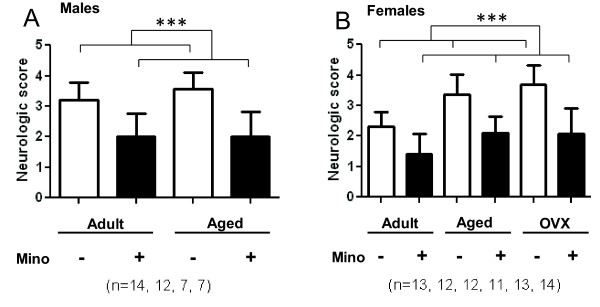
**Minocycline improves neurologic scores in males (A) and females (A)**. Neurologic evaluation was performed for surviving animals at 24 hours. Regardless of treatment adult females had significantly improved neurologic scores (both P < 0.0001, ***) than aged and OVX females who were not different than each other (P = 0.42). All data expressed as means ± SD.

We tested the neuroprotective efficiency of minocycline in two different groups of male mice: adult males (24.0 ± 4.6 weeks old) and aged males (18.1 ± 0.8 months old). The embolization lead to significant and stable drop of CBF in the MCA territory (18-21% of pre-ischemic values) as shown in Figures 2A and 2B. However, in adult males the occlusion was more stable (24% ± 4% of pre-ischemic values) for 6 hours than in aged group. We observed a modest spontaneous reperfusion in the aged males (41% ± 9% of pre-ischemic values for 6 hours after occlusion) that may resulted from the increased endogenous tPA activity in elderly [30,31]. Animals were randomized immediately after clot injection and treated with ether PBS (vehicle) or minocycline (6 mg/kg). Treatment with minocycline did not significantly affect regional CBF in aged males (Figure 2A), but induced modest spontaneous reperfusion in the adults (Figure 2B) at 6 hours (48% ± 8% of pre-ischemic values). Aged males had increased mortality at 24 hours post stroke that was markedly attenuated after minocycline treatment (Table 1). Minocycline significantly reduced the infarct volumes (P < 0.0001) and also improved functional outcomes (p < 0.0001) in surviving males in both adult and aged groups. Infarct volumes for adult males were reduced to 18.8% ± 14.5% in minocycline treated mice versus 43.1% ± 10.6% for the saline group, and treatment of aged group with minocycline decreased the infarct volumes to 16.8% ± 8.9% versus 34.6% ± 9.9% (Figure 4A). Neurologic scores at 24 hours post stroke in adult males were 2.0 ± 0.8 for the minocycline treated group versus 3.2 ± 0.6 for the saline group, and 2.0 ± 0.8 versus 3.6 ± 0.5 in the corresponding aged males (Figure 5A). Representative coronal sections of 24 hours post stroke brain are shown in Figures 2A and 2B.

### Acute minocycline treatment is neuroprotective for female mice

To analyze the effect of minocycline on the ischemic injury in female animals, we studied 3 groups of female mice: adult females (22.9 ± 3.3 weeks old), aged females (16.0 ± 1.1 months old), and ovariectomized (OVX) females (23.9 ± 2.7 weeks old). The age of adult and old females was selected based on reported time for maximal and stable cycle frequency (7-10 months) or onset of acyclicity (13-16 months) in C57BL/6 strain [26]. Consistent with the early report [26], we observed the cycle regularity in adult females (4-5 days) and significantly lengthened cycles in old females (7 - 11 days) with predomination of diestrus stage. Unlike other studies [5,24,25], ovariectomy was performed in early age at least 10 weeks before stroke onset. The estrous cyclicity test in OVX mice has confirmed their complete acyclicity and loss of estrogenic effect.

Consistent with cyclicity data the estrogen levels, determined at experimental endpoint, were 31.5 ± 51.0 pg/ml for adult females, 13.5 ± 9.5 pg/ml for aged females, and 6.6 ± 5.3 pg/ml for OVX females (Additional file 4, Figure S4). Estrogen levels were equivalent in OVX female mice and adult male mice (6.2 ± 0.7 pg/ml). All female mice were used regardless of cycle stage that should reflect the clinical scenario including both pre- and post menopause females in different estrogen cycle stages.

The degree of CBF reduction after embolization was similar among the groups (Figure 3). The degree of spontaneous CBF restoration was also similar among the female groups (39% - 51%), but was higher than in adult males due to possible enhanced endogenous fibrinolytic activity in females [32,33]. As expected, adult females were significantly more resistant to cerebral ischemic injury than males, but this advantage was abolished by aging or lack of estrogen in OVX females (P < 0.001). Mortality rates in the 24 hours studies were significantly higher in the aged and OVX groups compared to adults (P < 0.001, Table). Unlike aged animals, the mortality in OVX females was associated with intracranial post-stroke bleeding (Additional file 5, Figure S5). Minocycline treatment provided significant neuroprotection in all female groups. The mortality rates were markedly decreased in aged and significantly reduced in OVX females (P = 0.0061) attenuating bleeding. Moreover, the infarct volumes were reduced (Figure 4B; P < 0.0001) and neurologic scores were also improved (Figure 5B; P < 0.0001) in all minocycline treated females as compared to their corresponding vehicle treated controls.

### The inhibition of MMP-9 by minocycline

Because MMP-9 down-regulation has been previously shown as a potential mechanism of minocycline protection in young males [14,15], we investigated if minocycline down-regulates MMP-9 in females. All MMP-9 studies were performed by group blinded investigators. Consistent with previous reports [15] we did not observe the increase in MMP-9 level in vehicle treated animals of either group at 24 hours after stroke compared to sham controls (data not shown). In time dependent studies we detected the maximum MMP-9 level in the brain at 6 hours post stroke (Additional file 6, Figure S6). However, in plasma samples we detected low, or no, MMP-9 after ischemia compare to sham controls. This may be associated with using citrate to prevent blood clotting for plasma harvesting in our study.

**Figure 6:**
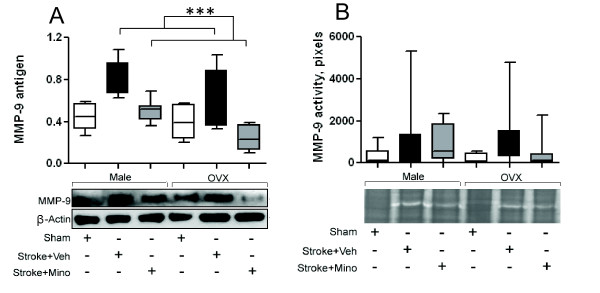
**Minocycline reduces level of MMP-9 in both genders**. A) Densitometric analysis of immunoreactive band intensities and representative Western Blot showing expression of 92 kDa-MMP-9 in ipsilateral hemispheres of adult male and OVX female mice (n = 6-7 animals per group) at 6 hours after thromboembolization. Values are expressed as relative intensity normalized to 42 kDa-β-actin intensity. The saline-treated mice had higher levels of MMP-9 than minocycline treated animals (P < 0.0001, ***). Minocycline and sham groups were not significantly different from each other (P = 0.77). B) Densitometric analysis of brain MMP-9 activity and representative zymography. The brain injury shows increased variability of MMP-9 activity relative to sham, but there are no sign differences between treatment or sex groups. All data expressed as means ± SD.

To estimate the gender effect, we used adult males and OVX females subjected to 6 hours stroke and randomized to sham-control, vehicle and minocycline treated (ns = 6-8) groups (Table 2). Figure 6A shows that stroke up-regulated brain level of MMP-9 protein and minocycline treatment reduced its expression in both genders (P < 0.0001). The vehicle-treated mice had significantly higher levels of MMP-9 protein than sham operated animals (P = 0.0007) and minocycline treated animals (P < 0.0001). No statistical difference between sham and minocycline groups were found (P = 0.77). Males had significantly higher level of MMP-9 expression than the OVX females (P = 0.0095). In the vehicle groups brain MMP-9 activity, as determined by zymography was highly variable in both genders (Figure 6B and Additional file 7, Figure S7). As shown in Additional file 7 (Figure S7), in a minority of animals acute ischemia did not result in up-regulation of MMP-9, such that no significant differences were found for sex or treatment.

**Table 2:** Mortality rates at 6 hours after stroke

Group	Treatment	Total in studies, n	Dead, n
Adult males	Vehicle	8	1
	Minocycline	7	0
	Sham	6	0

OVX females	Vehicle	6	0
	Minocycline	6	0
	Sham	6	0

### Mortality

Relative to saline, minocycline treatment significantly reduced mortality at 24 hours post-ischemia for OVX females (P = 0.006) and for aged mice (54% versus 25%, P = 0.037) (Table 1). There was not a difference in mortality (P = 1.0) for adult male and female mice (14% versus 16%). Collapsing across sex and age mortality was overall reduced in stroked mice with minocycline treatment compared to the non-minocycline controls (16% vs. 40%).

No mortality was observed in the 6 hour study (except one of the eight males in vehicle group, Table 2).

## Discussion

This study has several novel and important findings. First, we used a thromboembolic clot model rather than an intraluminal suture model (MCAO). Although MCAO, as the occlusion-reperfusion model, is widely utilized, the evolution of infarct within the territory of blood supplied by MCA is not been well explored [3]. Some study suggests that the fast reperfusion (at suture removal) may accelerate infarct development and modify the cellular mechanisms of the ischemic process [3,8,9]. A thromboembolic rodent model better mimics human stroke and has been previously described. Reperfusion is gradually and partially restored spontaneously but not until 6 to 12 hours when the ischemic cascade is already well advanced. This mimics the clinical scenario where the clot persists with slow spontaneous reperfusion and thrombolytic or mechanical reperfusion fails or is not performed. The model prevents the rapid evolution of the penumbra (as one of the limitations in clinical neuroprotective studies) and allows administration of a neuroprotective agent in the ischemic time windows that are suitable to mimic in clinical trials. Moreover, this represents the vast majority of human middle cerebral artery territory strokes (95% of stroke patients) where t-PA and mechanical removal are not given, or are ineffective.

Second, in this study we applied the novel approach to partially "humanize" the stroke mouse model. By supplementing the clot with human fibrinogen we adjusted the physiological level of fibrinogen in mouse blood clot (1.5 g/L) to the range of human normal value (1.5 - 4 g/L). This resulted in the increased strength and uniformity of the fibrin-rich clot and stabilized the occlusion. The stabilization of occlusion for 6 hours in adult males may be applied for further neuroprotective study beyond the window of thrombolytic therapy. The "humanization" of clot may also partially eliminate the cross species restriction barriers for binding of t-PA to the clot surface.

Third, to the best of our knowledge, this is the first study that tested minocycline in an thromboembolic stroke model to investigate gender and age-dependent influences on stroke injury and outcomes. Our novel findings provide evidence that minocycline was effective at reducing infarct size and improving short-term neurological outcome in young male and female mice, OVX female mice and aged male and female mice.

Overall, analyzing all subgroups and reflecting to the clinical situation, minocycline reduced mortality (16% vs. 40%), decreased the infarct size (13.3% ± 1.4% vs. 32.8 ± 1.7%, P < 0.0001, Additional file 8, Figure S8A) and improved neurological outcomes (1.9% ± 0.1% vs. 3.2 ± 0.1%, P < 0.0001, Additional file 8, Figure S8B).

Thus, this is the first comprehensive attempt to study minocycline's effect by both sex and age. Using the thromboembolic stroke model we found that minocycline protected not only young adult male but also female and aged (male & female) brains compared to control vehicle treatment.

This study is also novel in the context of the revised preclinical STAIR criteria call for testing of neuroprotective agents in female mice and aged mice. Most experimental stroke studies have been done exclusively in young male animals, although stroke mainly affects the elderly. Only a few studies have used female or aged rats, particularly with an embolic clot model [11,34,35]. Moreover, we could find no comprehensive reports of the thromboembolic clot model in aged or female mice. One reason may be the high mortality in this model in aging animals. In our model we found a mortality of nearly 50% in both aged females and males and a similar high mortality in OVX females. Minocycline significantly reduced this mortality in OVX females and aged mice. Further studies are needed and planned to determine the effect of t-PA in the embolic clot model in younger and older female mice and the expansion of this time window for minocycline treatment. Including animals of both sexes and aged animals in an embolic clot model is warranted for design of future clinical trials.

It is interesting that in MCAO preclinical model minocycline was neuroprotective in male mice but not in recently OVX females [24]. While minocycline is a potent PARP inhibitor at nanomolar concentrations [5,22], minocycline acts by multiple mechanisms of action [13,14,16,18,19], one of which is MMP-9 inhibition [14,23]. Activation of MMP-9 plays an important role in mediating tissue injury during human ischemic stroke and is associated with ICH after t-PA [36-40]. Suppression of MMP-9 may lead to safer therapeutic outcomes in acute stroke [41]. Although the different mechanisms may be responsible for neuroprotection with minocycline, the difference in stroke models and following ischemic sequences may be the key factors of attenuation or augmentation of the particular mechanism. Here, we demonstrated that brain MMP-9 was up-regulated in an embolic model after ischemia in both male and female mice. Moreover, minocycline reduces MMP-9 expression for both sexes. MMP-9 activity has been shown to be up-regulated in the blood and brain of ischemic rodents, but all studies to date have used male rodents. MMP-9 activity was highly variable following ischemia in this model and others [15,41] when t-PA is not given. While there appeared to be a trend towards less variability of MMP-9 brain activity with minocycline treatment we did not detect a significant overall reduction in activity.

### Study limitations

This work presents the proof of principle study to demonstrate the neuroprotective effect of minocycline in females using the thromboembolic stroke model. The study was designed with administration of minocycline right after stroke onset and a single endpoint at 24 post-stroke hours, which may limit the translational potential. However, in light of the novel pre-clinical model for minocycline evaluation, our data reveal that minocycline is an efficient neuroprotective agent in females and aged animals. Further studies are needed to address later endpoints and neuroprotective pathways of minocycline.

## Conclusions

In summary, the thromboembolic model in mice provided evidence of neuroprotection with minocycline in both sexes and in older mice. This model could be used as a tool for the stroke researcher to design future experiments in the attempt to translate stroke therapy in the clinical practice.

## Competing interests

The authors declare that they have no competing interests.

## Authors' contributions

MNH conceived and designed the study; carried out the stroke surgery and neurological scoring; helped in the data analysis and interpretation; helped to draft the manuscript. WL carried out the infarct assessment and analysis. AA carried out immunoassay; performed the densitometry blot analysis; helped with neurological scoring and tissue harvesting. MAZ carried out the zymography assay of plasma samples; helped with the data analysis. SO carried out the zymography assay of tissue samples; carrier out the zymography blots analysis and helped in interpretation. MHJ performed the statistical analysis; made critical revision of the manuscript. AE participated in the study design and coordination; helped with the zymography analysis; helped to revise the manuscript; handled funding and supervision. WDH participated in the study design and coordination; participated in the data analysis; helped to revise the manuscript; handled funding and supervision. DCH conceived and designed the study; helped in the study coordination; analyzed and interpreted the data; helped to draft the manuscript and made critical revision of the manuscript; handled funding and supervision. IYS conceived, designed and coordinated the study; analyzed and interpreted the data; drafted and revised the manuscript; handled funding and supervision. All authors read and approved the final manuscript.

## Supplementary Material

Additional file 1 **Additional Figure 1, (Figure S1)**. Representative PeriScan scanning imaging of brain at 1 hour after stroke (PeriScan PIM 3 System, North Royalton, Ohio). Click here for file

Additional file 2 **Additional Figure 2 (Figure S2)**. Representative PeriScan scanning imaging of brain at 24 hours after stroke (PeriScan PIM 3 System, North Royalton, Ohio). Click here for file

Additional file 3 **Additional Figure 3 (Figure S3)**. Representative images of infarct volume determined by T2 diffusion-weighted MRI. Click here for file

Additional file 4 **Additional Figure 4 (Figure S4)**. Comparison of estrogen level in females mice (data are presented in the Results). Click here for file

Additional file 5 **Additional Figure 5 (Figure S5)**. Representative brain images of OVX females treated with vehicle and minocycline. Click here for file

Additional file 6 **Additional Figure 6 (Figure S6)**. Densitometric analysis (A) and representative Western Blots (B) of time-dependent MMP-9 expression in brain. Click here for file

Additional file 7 **Additional Figure 7 (Figure S7)**. Representative zymography of MMP-9 activity. Click here for file

Additional file 8 **Additional Figure 8 (Figure S8)**. Summarized analysis of minocycline to reduce infarct (A) and improve neurological outcomes (B) after acute ischemia. Click here for file

Additional file 9 **Additional Figure Legends**. Legends for Additional Figures. Click here for file

Additional file 10 **Additional Methods**. Measurement of cerebral perfusion and MRI procedure. Click here for file
