# Supplementary material for: Sex-independent neuroprotection with minocycline after experimental thromboembolic stroke
Source: Exp Transl Stroke Med. 2011 Dec 16;3:16. doi: 10.1186/2040-7378-3-16 (PMC3287111; doi:10.1186/2040-7378-3-16)
Supplement: Additional file 1 — Additional Figure 1, (Figure S1). Representative PeriScan scanning imaging of brain at 1 hour after stroke (PeriScan PIM 3 System, North Royalton, Ohio). [file 2040-7378-3-16-S1.PDF]

Calculations

Item	Min	Max	Std.dev	Mean	Change	% Change in CBF	Median	mm2	Sites	SNR	Time
**Image 1**											0:00:00
**Contra**	40.8	2789.8	594.7	851.7	ref	Ref: Contralateral side	699.7	42	156	67.7	0:00:00
**Ipsilateral**	-19.1	1491.8	229.9	253	-598.8	-70.3	209.9	30.4	113	17	0:00:00
**Image 2**											0:00:53
**Contra**	-30.7	2975.1	674.3	837	ref	Ref: Contralateral side	628.6	36.6	136	67.5	0:00:53
**Ipsilateral**	-14.1	1984.7	262.7	250.5	-586.5	-70.1	194.5	30.4	113	17.8	0:00:53
**Image 3**											0:01:46
**Contra**	-4.6	2748.1	657.8	819.8	ref	Ref: Contralateral side	660.5	36.6	136	66.2	0:01:46
**Ipsilateral**	-20.5	1685.5	244.1	234.8	-585	-71.4	172.6	30.4	113	16.4	0:01:46

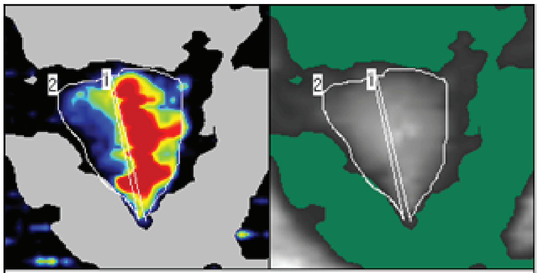

Image 1
00:00:00

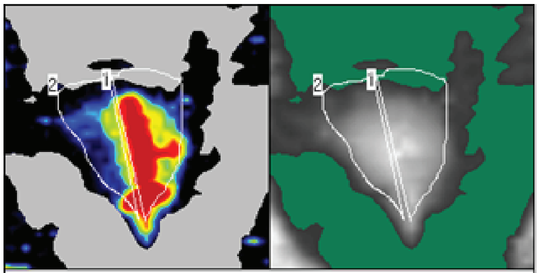

Image 2
00:00:53

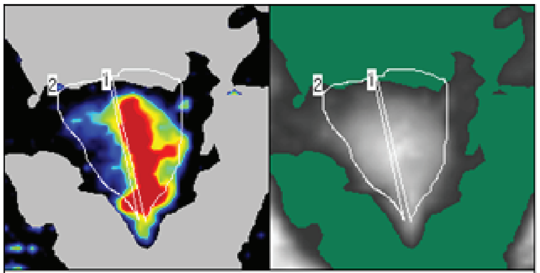

Image 3
00:01:46
