# Supplementary material for: Sex-independent neuroprotection with minocycline after experimental thromboembolic stroke
Source: Exp Transl Stroke Med. 2011 Dec 16;3:16. doi: 10.1186/2040-7378-3-16 (PMC3287111; doi:10.1186/2040-7378-3-16)
Supplement: Additional file 2 — Additional Figure 2 (Figure S2). Representative PeriScan scanning imaging of brain at 24 hours after stroke (PeriScan PIM 3 System, North Royalton, Ohio). [file 2040-7378-3-16-S2.PDF]

Calculations

Item	Min	Max	Std.dev	Mean	Change	% Change in CBF	Median	mm2	Sites	SNR	Time
**Image 1**											0:00:00
**Contra**	54.7	1326.6	281.8	693.5	ref	**Ref: Contralateral side**	683.7	20.9	75	37.7	0:00:00
**Ipsilateral**	19.7	1367.7	278.3	323.5	-370	**-53.4**	243.6	25.4	91	17.4	0:00:00
**Image 2**											0:00:53
**Contra**	40.8	1546.5	283.5	687.6	ref	**Ref: Contralateral side**	671.7	20.9	75	37.3	0:00:53
**Ipsilateral**	13.6	1047.4	256.1	307.5	-380.2	**-55.3**	231.1	25.4	91	16.3	0:00:53
**Image 3**											0:01:46
**Contra**	79.3	1337.5	267.2	678	ref	**Ref: Contralateral side**	661.9	20.9	75	36.5	0:01:46
**Ipsilateral**	25.3	1270.9	260.1	309.6	-368.4	**-54.3**	228.9	25.4	91	16.2	0:01:46

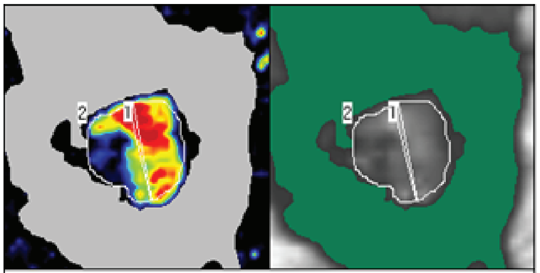

Image 1
00:00:00

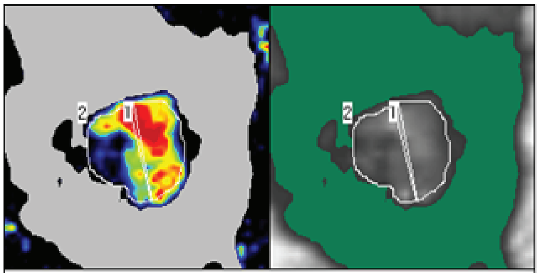

Image 2
00:00:53

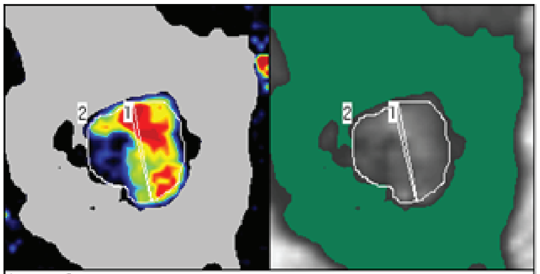

Image 3
00:01:46
