# Supplementary figures and images for: Sex-independent neuroprotection with minocycline after experimental thromboembolic stroke
Source: Exp Transl Stroke Med. 2011 Dec 16;3:16. doi: 10.1186/2040-7378-3-16 (PMC3287111; doi:10.1186/2040-7378-3-16)

A

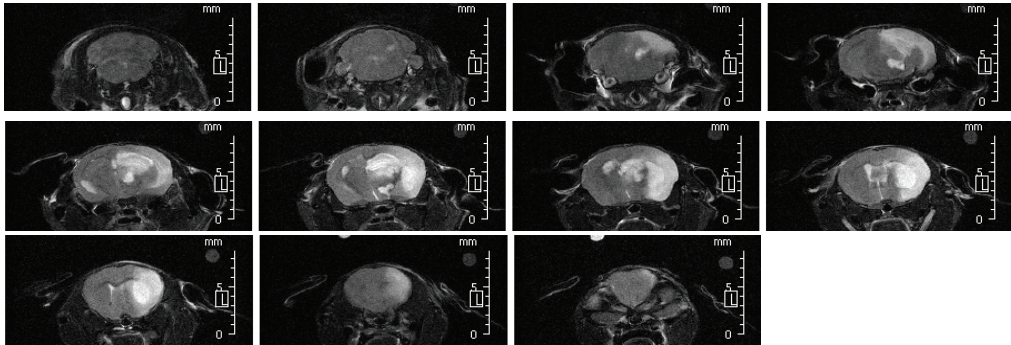

B

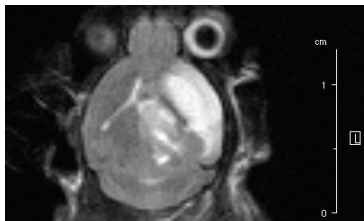

C

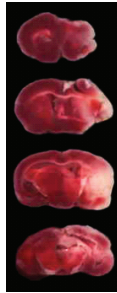

D

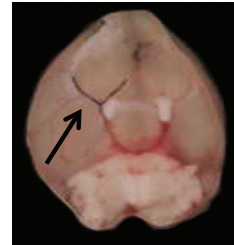

Supplement: Additional file 3 — Additional Figure 3 (Figure S3). Representative images of infarct volume determined by T2 diffusion-weighted MRI. [file 2040-7378-3-16-S3.PDF]

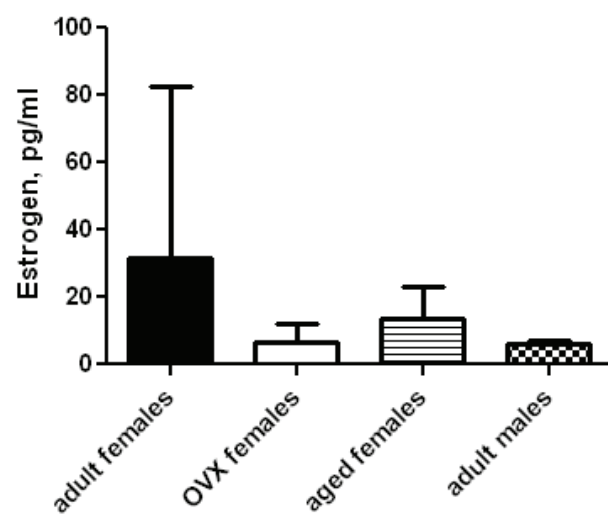

Supplement: Additional file 4 — Additional Figure 4 (Figure S4). Comparison of estrogen level in females mice (data are presented in the Results). [file 2040-7378-3-16-S4.PDF]

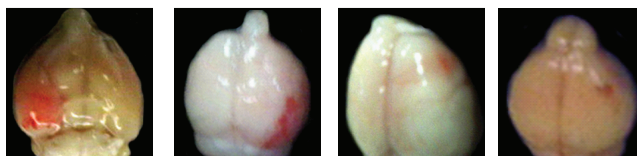

Vehicle

Minocycline

Supplement: Additional file 5 — Additional Figure 5 (Figure S5). Representative brain images of OVX females treated with vehicle and minocycline. [file 2040-7378-3-16-S5.PDF]

**A**

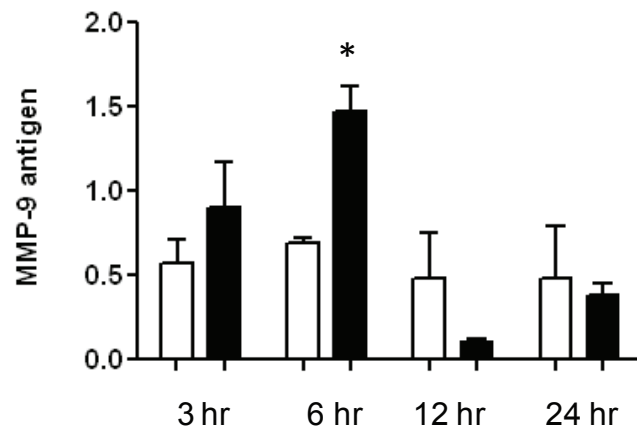

**B**

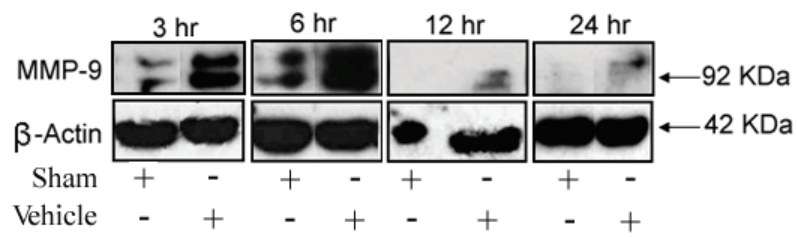

Supplement: Additional file 6 — Additional Figure 6 (Figure S6). Densitometric analysis (A) and representative Western Blots (B) of time-dependent MMP-9 expression in brain. [file 2040-7378-3-16-S6.PDF]

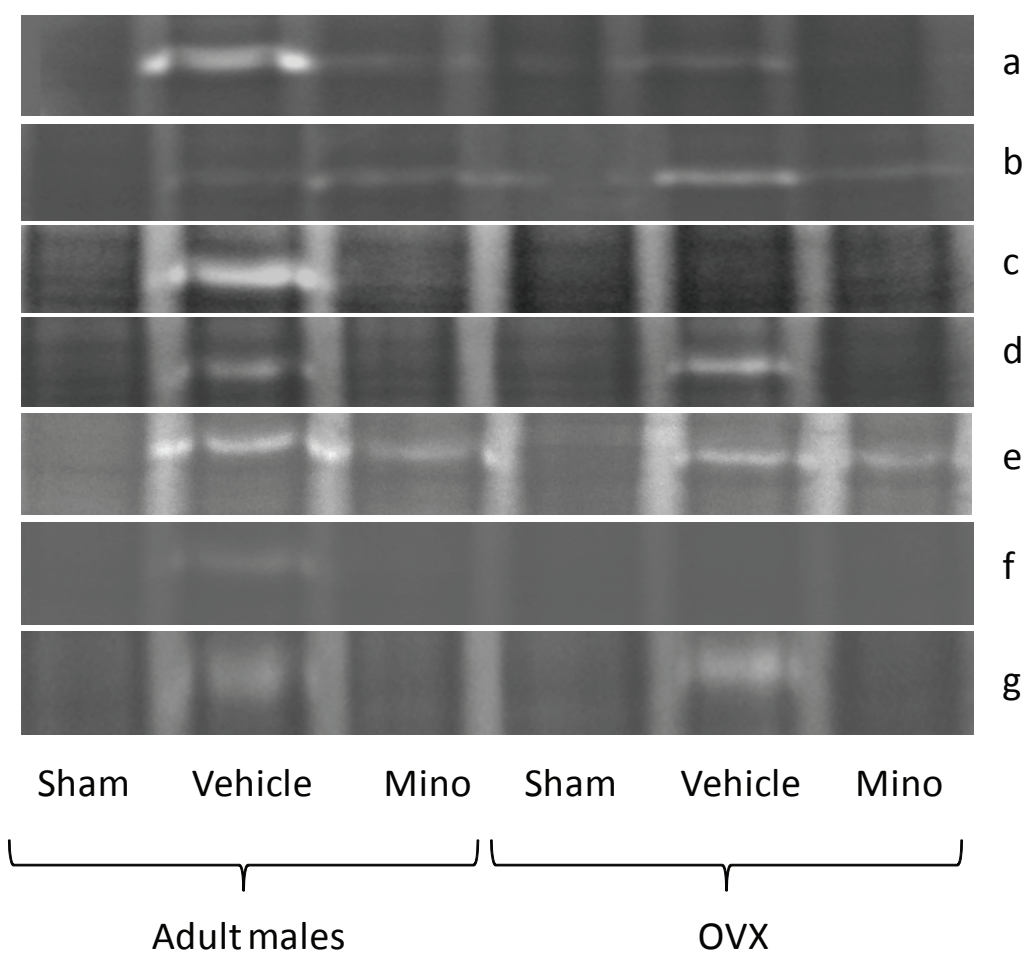

Supplement: Additional file 7 — Additional Figure 7 (Figure S7). Representative zymography of MMP-9 activity. [file 2040-7378-3-16-S7.PDF]

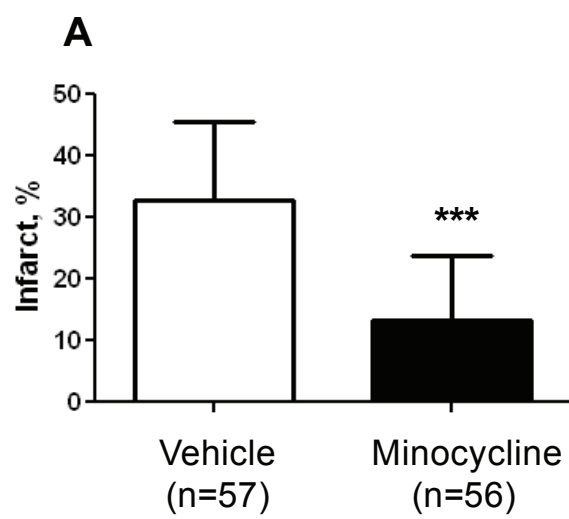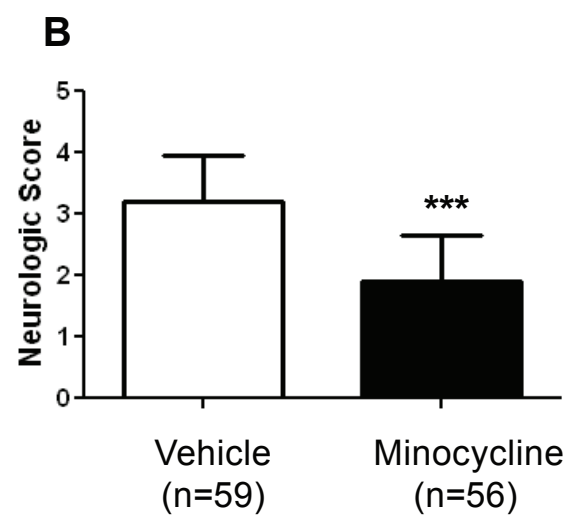

Supplement: Additional file 8 — Additional Figure 8 (Figure S8). Summarized analysis of minocycline to reduce infarct (A) and improve neurological outcomes (B) after acute ischemia. [file 2040-7378-3-16-S8.PDF]
