# Supplementary material for: Sex-independent neuroprotection with minocycline after experimental thromboembolic stroke
Source: Exp Transl Stroke Med. 2011 Dec 16;3:16. doi: 10.1186/2040-7378-3-16 (PMC3287111; doi:10.1186/2040-7378-3-16)
Supplement: Additional file 9 — Additional Figure Legends. Legends for Additional Figures. [file 2040-7378-3-16-S9.DOC]

**Additional Figure Legends**

**Additional files 3 and 4.** Representative images of scanning laser Doppler imaging system (PeriScan PIM 3 System, North Royalton, Ohio) and analysis of cerebral perfusion in an adult male. The image illustrates the percentage of CBF change in ischemic versus contralateral hemisphere at 1 hour (Additional file 3) and 24 hours (Additional file 4) after thromboembolization of a representative adult male mouse. The CBF decline was to 29.4% of baseline after 1 hour and 45.7% of baseline after 24 hours.

**Additional file 5.** Infarct volume in a representative adult male at 24 hours as detected by coronal T2 diffusion-weighted MRI (A), axial T2 diffusion-weighted MRI (B), and corresponding coronal sections staining with 2,3,5-triphenyltetrazolium chloride (C). The ipsilateral MCA territory of ischemic brain is shown (D), and the clot site is indicated by an arrowhead.

**Additional file 6.** Analysis of estrogen level in females mice. Plasma samples of adult, aged and OVX female mice were subjected to estrogen enzyme immunoassay and compared to adult males. Data expressed as means ± SD.

**Additional file 7.** Minocycline prevented post-stroke hemorrhage in OVX female mice. Representative images of OVX female brain treated with vehicle (left) and minocycline (right). Mice were euthanized at 24 hours after stroke for injury assessment. Brains were perfused with ice cold phosphate-buffered saline, and images were assessed immediately before coronal slicing for TTC analysis.

**Additional file 8.** Densitometric analysis of immunoreactive band intensities (A) and representative Western Blots (B) showing time-dependent MMP-9 expression in brain. Adult male mice were subjected to thromboembolic stroke or sham surgery and sacrificed at various post-stroke times (3-24 hours). The tissue of ipsilateral hemispheres was homogenized and analyzed to MMP-9 expression compare to β-actin. Values (mean ± SD) are expressed as relative intensity normalized to corresponding 42 kDa-β-actin intensity. The maximal MMP-9 expression was detected at 6 hours after stroke onset.

**Additional file 9.** Representative zymograpgy blots showing MMP-9 activity in ipsilateral hemispheres of adult male and OVX female mice at 6 hours after thromboembolization.

**Additional file 10.** Effect of minocycline to reduce infarct (A) and improve neurological outcomes (B) after acute ischemia. All experimental subgroups were combined to determine benefits of minocycline treatment compared with vehicle to reflect the clinical situation. The statistical analysis was performed using unpaired two-tailed t-test. Data presented as mean ± SD. *** P<0.0001.
