# Supplementary material for: Sex-independent neuroprotection with minocycline after experimental thromboembolic stroke
Source: Exp Transl Stroke Med. 2011 Dec 16;3:16. doi: 10.1186/2040-7378-3-16 (PMC3287111; doi:10.1186/2040-7378-3-16)
Supplement: Additional file 10 — Additional Methods. Measurement of cerebral perfusion and MRI procedure. [file 2040-7378-3-16-S10.DOC]

**Additional Methods**

**Measurement of cerebral perfusion**

The cerebral perfusion was measured by the scanning laser Doppler imaging system (PeriScan PIM 3 System, North Royalton, Ohio) as described previously [29]. The scanner was positioned to scan a 1.5 × 1.5 cm area (1600 detection points) covering the cross-point of the coronal and sagittal sutures. In this system, a built-in photo detector detects the reflected light from moving blood cells within 0.5 cm of the cortical surface, and a color-coded image is acquired based on the concentration and mean velocity of these blood cells using the LDPIwin software (Perimed, North Royalton, Ohio). Data are presented in Additional file 3 and additional file 4.

**MRI**

To confirm the ischemic lesson, T2 diffusion-weighted MRI was performed on representative adult male mice at 24 hours after stroke using a horizontal 7.0 T BioSpec MRI spectrometer (Bruker Instruments, Billerica, MA) equipped with equipped with a 8.9-cm micro imaging gradient insert (100 gauss/cm). All T2-weighted MRI procedures were performed by Core Imaging Facility for Small Animals (CIFSA) at GHSU.

Data are presented in Additional file 5.
